# Evaluation of rapid detection methods for H5N1 virus using biosensors: An AI-based study

**DOI:** 10.6026/9732063002001516

**Published:** 2024-11-30

**Authors:** Roberto Eggenhöffner, Paola Ghisellini, Cristina Rando, Simonetta Papa, Allen khakshooy, Luca Giacomelli

**Affiliations:** 1 Department of Surgical Sciences and Integrated Diagnostics (DISC), University of Genova, Corso Europa 30, Genova - 16132, Italy; 2 Polistudium SRL, Milan, Italy; 3 Department of Internal Medicine, Valley Hospital Medical Center, Las Vegas, NV - 89106, USA

**Keywords:** H5N1 virus detection, biosensors, lateral flow, field-effect transistor, quartz crystal microbalance

## Abstract

High mortality and zoonotic potential predispose the H5N1 avian influenza virus as a critical threat. knowing that an epidemic could be occurring, quick and precise diagnostic techniques are essential for managing and containing possible epidemics. To detect H5N1 in saliva samples, this study investigates the theoretical design, simulation and evaluation of three kind of biosensors based on different technologies with potential as rapid identifications tools to diagnose quickly H5N1: Lateral Flow Tests (LFT), Field Effect transistors (FET) based electrochemical sensors and Quartz Crystal Microbalance (QCM) sensors. Through detailed AI-based simulations, we show the capabilities, sensitivities and specificities of these biosensors, highlighting their potential for applications in general biology as well as their suitability both for routine home practice and for applications by control entities in public settings. We therefore wish to pave the way to a framework for the quick creation of detection tools that can be swiftly implemented for rapid deployment in case of an outbreak of disease.

## Background:

H5N1 is an avian virus, whose mutated variants circulates and spreads amongst mammals. The influenza caused by this virus is becoming a serious public health threat that can lead to severe respiratory illness and death, in animals and in humans [1, 2-3]. The key to managing viral outbreaks that may have epidemic or pandemic potential is early identification and fast diagnosis, as well as precluding further spread. Case in point, the Corona Virus Disease-2019 (CoViD-19) pandemic has made evident the need for rapid and reliable diagnostic tools to screen, diagnose and manage viral infections [4]. Technologies such as lateral flow tests (LFTs) and electrochemical sensors, adopted to face the spread of previous pandemics such as CoViD-19, played pivotal roles in the quick identification and isolation of infected individuals in the last years. Accordingly, this study describes the development of computer simulations and testing of LFT, Field Effect transistors (FET)-based electrochemical and Quartz Crystal microbalance (QCM) diagnostic sensors for H5N1 detection with an emphasis on rapid preparedness for deployment in case of a H5N1 epidemic/pandemic outbreak. Gold Nanoparticle-based LFT act as ideal biosensors for quick and sensitive assessment of viral nucleic acids. A Gold Nanoparticle-based LFT was also established for the detection of fish nervous necrosis virus [5-6]. The choice of a FET biosensor is recommended because it inherently permits an ultra-high response speed [7]. There were few advanced versions developed like graphene-based/MoS2/Silicon nanowires exhibited high sensitivity for SARS-CoV-2 and other viral nucleic acids detection. The biosensors based on the QCM are introduced by coating antibody onto plates to detect viral proteins and whole intact viruses with high specificity. For example, a QCM biosensor was developed for detecting antibodies against the African swine fever virus with high sensitivity [8]. In other settings, aptamer-based QCM was adopted for the sensitive detection of leukemia cells, demonstrating potential for viral detection through specific nucleic acid binding [9]. AI-based approaches have a mounting role in the development of computer simulations, which guide researchers and decision-makers in building hypotheses based on a stronger scientific foundation. This methodology has been applied in various fields and biosensors are no different [10]. Our focus characterized the detection of H5N1 in saliva samples, which offer a less invasive and more practical means of sample collection for diagnosis. By simulating the performance of these biosensors at various virus concentrations, we showed here their potential capability for actual applications in the context of viral infections and emphasize the importance of being prepared with advanced diagnostic tools to quickly respond to emerging viral threats. A comparison between LFT for H5N1 with the LFT for SARS-CoV-2 is also provided.

## Methods:

## Development of lateral flow tests (LFT):

The test strip is composed of an H5N1 virus-specific capture antibody-labeled conjugate zone, a test zone with anti-species antibodies to verify the test's validity and a sample pad collecting saliva on a nitrocellulose membrane. Monoclonal antibodies are used to detect H5N1 targeting the hemagglutinin (HA) and neuraminidase (NA) proteins. Saliva samples prepared to express known concentrations of H5N1 from the lowest to highest values are placed on the sample pad for the generation of a standard curve. In the conjugate zone, the unknown sample interacts with the labeled antibodies as it migrates through the membrane via capillary action. A color shift in the test and control lines is used to achieve visualization; the test zone shows the presence of H5N1 and the control zone verifies the test's functionality [11]. The study modeled the optical density (OD) response of LFT biosensors for detecting salivary H5N1 virus particles. The migration of samples through the membrane plate was modeled using the Lucas-Washburn equation, incorporating parameters such as surface tension, pore radius and viscosity. The antibody-antigen interaction in LFT was modeled by the Langmuir adsorption model, with specific association and dissociation rate constants for H5N1 antibodies.

## Development of FET-based electrochemical sensors:

The FET-based sensor exploits the unmatched electrical properties of graphene as the substrate material in semiconducting devices. Gold electrodes are deposited on the graphene to serve as the source and drain, while the gate is functionalized with monoclonal HA antibodies specific to H5N1. Thiol groups are used to attach the antibodies to the graphene gate. Saliva samples are applied to the functionalized gate, where the binding of H5N1 to the antibodies causes a change in the electrical properties of the graphene. This change is measured as a variation in the current (ΔI) using a potentiostat, providing a quantitative indication of the virus concentration. The sensitivity of the FET sensor allows detecting concentrations as low as 10 particles/mL the lowest limit [12].

## Development of quartz crystal microbalance (QCM) sensors:

QCM sensors are based on the principle that a change in mass on the surface of a vibrating quartz crystal affects its resonant frequency. For H5N1 detection, the quartz crystal is coated with monoclonal antibodies specific to the HA and NA proteins. As H5N1 particles bind to these antibodies, the added mass causes a measurable change in the crystal's frequency. The QCM sensor setup involves preparing the quartz crystal with a thin gold layer, onto which antibodies are immobilized using self-assembled monolayers (SAMs) of thiols. Saliva samples are applied to the sensor and the binding of H5N1 virus, when present, produces a resulting frequency shift that can be monitored with high accuracy using a frequency counter. In turn, this method provides a direct and quantitative measurement of the virus concentration [13]. Mass changes due to virus adsorption on the functionalized gold surface are measured as frequency shifts. Mass and frequency variations are related throughout the following Sauerbry's well-known equation [14].

Where Δf is the frequency change, Δm the mass change, f0 the fundamental frequency of the quartz crystal (5 or 10 MHz usually), A the area of the gold electrode on the crystal plate (typical value 0.2 cm^2^), ρ the density of quartz plate (2.65 g/cm^3^), µ the shear modulus of quartz (2.95 x 10^11^ g/cms^2^).

## Simulations of detections with the three biosensors:

To evaluate the performance of the developed biosensors, we conducted AI-based simulations considering different concentrations of H5N1 in saliva samples and their respective limits of detection (LOD). Table 1 summarizes the approach used for each simulation. In the case of SARS-CoV-2, the median viral load in posterior oropharyngeal saliva or other respiratory specimens at presentation was approximately 5.2 log_10_ copies/mL, with an inter quartile range (IQR) of 4.1-7.0 log_10_ copies/mL [15]. The average PCR cycle threshold values ranged from were 29 to 31 log_10_ copies/ml for symptomatic and asymptomatic cases respectively [16]. Fewer specific data on salivary viral load are available for H5N1, compared to SARS-CoV-2. However, similar methods of detection using saliva samples were suggested based on respiratory tract sampling and antibody presence [17]. It follows that the three sensors considered in the present work should be expected to measure virus concentrations ranging from 10^4.1^ to 10^7.0^ units/mL accordingly to kim *et al.* 2020 [18].

## Results:

Simulations performed in the reported range confirmed the concentration detection limits (*i.e.*, LOD) 10^4.1^ to 10^7.0^ units/mL. We report below the change in color intensity/current/frequency measured to determine the sensor's response to varying concentrations of H5N1.

## Simulation of a lateral flow test (LFT) for H5N1 in saliva:

**Figure 1:**
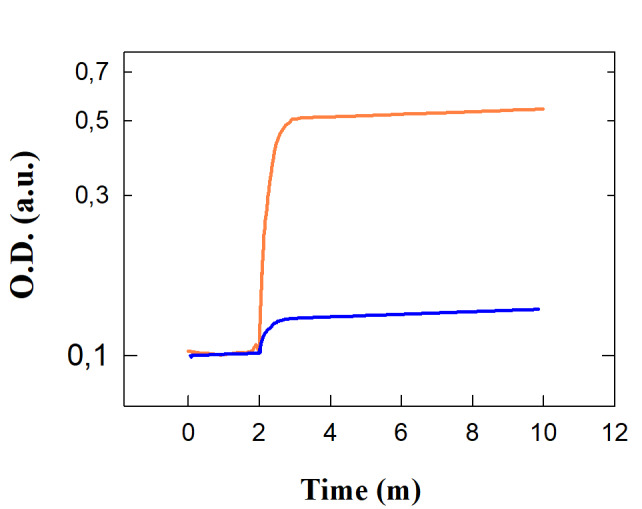
Color Intensity Response (OD) vs time in minutes of a LFT biosensors to Different Concentration of H5N1 Virus Particles in Saliva (blue: low, orange: high) from the simulation model. Curve blue: low concentration response; curve orange: high concentration response.

The simulation for the LFT involves testing H5N1 concentrations in saliva at the limiting levels: low (10^4^ particles/mL) and high (10^7^ particles/mL), with LOD at 10^4^ particles/mL. The optical density response was modeled over time, considering delays before visibility for low (10^4^ particles/mL) and high (10^7^ particles/mL) concentrations (Appendix 1). The results showed a significant optical density increase after 5 minutes for low concentrations and 2 minutes for high concentrations. Enhanced scaling factors ensured detectability even at low viral loads. Given the human retina's logarithmic response to light, the optical density responses were visualized on a logarithmic scale for clear comparison. At low concentrations near the detection limit, a slight discoloration in the test zone is expected, which produces a faint but discernible stain on the optical response chart. At high concentrations, much above the detection limit, the test zone exhibits an intense and saturated stain, which indicates a high viral load. These results predict the LFT's capability for reliable initial screening correlating with virus concentration. Figure 1 illustrates the optical density response of an LFT biosensor over time for the above concentration limits of salivary H5N1 virus particles. At low concentration (10^4^ particles/mL, blue curve), optical density increases slightly and then stabilizes at a lower intensity, indicating low viral particle detection. At high concentration (10^7^ particles/mL, orange curve), the response shows a rapid and significant increase, stabilizing at a higher intensity value. Taken together, this signifies high viral particle detection, with a strong signal maintained after the initial increase. Overall, the comparison of the two curves in Figure 1 shows that the sensor can produce discernible signals at various viral particle concentrations. The response is faster and noticeably more intense at high concentrations and slower and less intense at low concentrations. The differentiation seen is essential for precise viral load monitoring and detection in diagnostic applications.

## Simulation of a FET-based electrochemical biosensor for H5N1 detection in saliva:

The graphene-based field-effect transistor (FET) or metal-oxide-semiconductor field-effect transistor (MOSFET) sensor consists of a graphene channel, gate dielectric, source and drain electrodes and a passivation layer. Both FET and MOSFET are types of transistors that control the flow of current between the sources and drain electrodes via an electric field applied to the gate. In these devices, the graphene channel, this is a single-layer graphene sheet, acts as the conductive path between the sources and drain electrodes. The gate dielectric, a combination of SiO_2_ and graphene oxide, provides the necessary capacitance to control the channel conductivity. The detection mechanism involves the binding of H5N1 particles to specific receptors (antibodies of H5N1) on the graphene surface, which changes the surface charge density Δσ. This binding event modulates the gate voltage (VGS), thereby affecting the channel conductivity. The change in surface charge density due to the binding event results in a measurable change in the drain current (ΔID), which can be converted to a voltage through an amplification stage. The simulations of these sensors are performed at the same concentration levels as the above LFT, *i.e.* 10^4^ particles/mL and 10^7^ particles/mL concentration values. The FET sensor's limit of detection limits (LOD) ranges from 10 to 10^3^ depending on experimental choices [12]. The change in charge density Δσ are 1.60x10^-9^ C/cm^2^ and 1.60x10^-6^ C/cm^2^ for low and high concentration limits, respectively. The carrier mobility µ is very high in graphene, of the order of 5000 cm^2^/V.s that was observed to reduce in heavily deposited surfaces, as discussed in Appendix 2. The main equation that relates the change in drain current (ΔID) to the relevant physical parameters of the FET biosensor is as follows:

ΔID= µ VDS Δσ (W/L)

**Figure 2:**
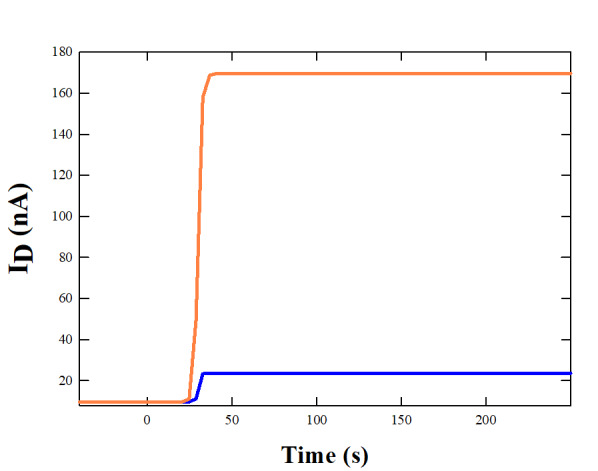
Change in drain current (ΔID) over time for graphene-based FET/MOSFET sensors exposed to different concentrations of H5N1 particles. The blue line represents the response to a low concentration of 10^4^particles/mL, showing a significant change in current at the reaction time and stabilizing at approximately 24 nA, 10 nA above baseline. The orange line represents the response to a high concentration of 10^7^particles/mL, with a rapid increase in current, indicating the sensor's sensitivity and potential saturation at higher concentrations. The dashed gray line represents the baseline current in the absence of H5N1 particles.

Where VDS is the drain-source voltage, W and L are the width and length of the channel, respectively. The equation adopted in the simulations shows that the change in current is directly proportional to the applied drain-source voltage, the change in charge density due to viral particle binding and the aspect ratio of the graphene channel. This relationship helps in understanding how sensitive the sensor is to changes in viral particle concentration and how it translates into measurable electrical signals. The simulated ΔID current at the selected low and high concentrations, as shown in Figure 2, starts rising at the initial reaction time and quickly reaches stable saturation values above the baseline, the detection limit (LOD) of the sensor. This response reflects the possible sensor's detection of low concentration of viral particles, with the signal stabilizing at a low but detectable current value. The rapid rise indicates the FET sensor's high sensitivity and quick response both for high and also low levels of viral particles. The baseline current is the value predicted in absence of external charges linked to surface antibodies. The ΔID response at high concentrations of viral particles shows the sensor's ability to handle high concentrations and produce a strong signal, highlighting its suitability for detecting high viral loads. A graphene-based FET/MOSFET exhibits a good sensitivity also at low viral concentrations, resulting in a significant change in drain current, crucial for early detection of H5N1 particles in saliva.

## Simulations for the quartz crystal microbalance (QCM) biosensor:

**Figure 3:**
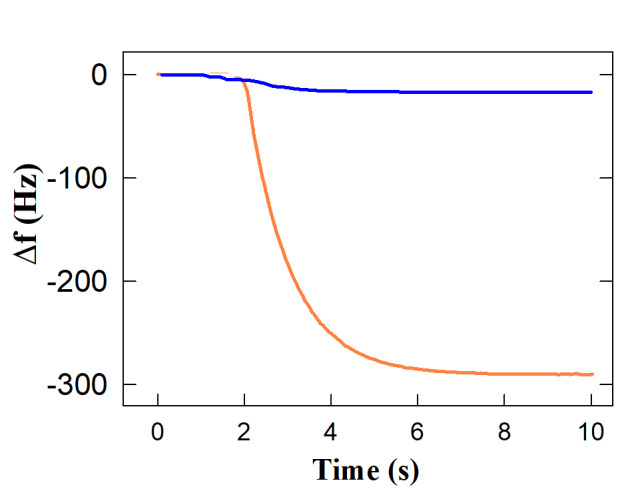
Frequency decreases of the QCM Biosensor to Different Concentrations of H5N1 Virus Particles in Saliva (blue: low concentration, orange: high concentration)

In order to quantitatively simulate the performance of a QCM biosensor for detecting salivary H5N1, we must focus on the relationship between virus concentration and frequency shifts. In our study, we calculated the frequency shifts for a QCM biosensor to detect salivary H5N1 at two concentration levels: low (10^4^ particles/mL) and high (10^7^ particles/mL). For the low concentration, the number of particles is approximately 500 per 50-microliter. Given that each H5N1 particle has a mass of approximately 100 femtograms (fg), the total mass added to the sensor is 5 x 10^-11 grams. This results in a frequency shift of approximately -2.9 Hz, which, although small, is within the practical measurement capability for QCM sensors. For the high concentration of 10^7^ particles per milliliter, the number of particles per 50-microliter drop sample is approximately 5x10^5^. The total mass added to the sensor is thus 5x10^-8^ grams, resulting in a frequency shift of approximately -290 Hz. This significant frequency shift is well within the measurable range of QCM sensors, indicating in this case the very high presence of H5N1. The above findings, displayed in Figure 3, show that the QCM sensor can accurately quantify salivary H5N1 concentrations and produce detectable frequency shifts that match the virus load. The sensor showcases its potential utility in detecting high viral loads in clinical samples by effectively identifying significant changes at higher concentrations.

## Discussion:

## Performance and potential applications of the three sensors:

Through detailed computer simulations, we showed the capabilities, sensitivities and specificities of three different biosensors (LFT, FET and QCM) for the detection of H5N1 in saliva samples. Our data show that salivary H5N1 can be quickly and affordably detected using LFT. The easy-to-use nature and rapid results make LFTs is appropriate for both field settings and large-scale screenings. The sensitivity of the LFT is limited at lower viral loads and its dynamic range is effective within a narrow concentration window from 10^4^ to 10^7^ particles/mL. Thus, it provides qualitative results and therefore is a highly valuable tool for initial screenings without any discomfort to the tested subject. LFT may also be of great use for extensive screening in less-developed countries [3] because it is a practical protocol that is inexpensive and that requires little training and no complex equipment. Widespread testing is possible even in remote locations because they can be conducted by healthcare professionals with only rudimentary laboratory experience. Additionally, in situations where prompt diagnosis can have a substantial impact on the management and containment of infectious diseases, the quick results that LFTs provide can be lifesaving. LFTs are especially well-suited for use in field operations and mobile clinics due to their portability and simplicity, which makes mass screenings during outbreaks easier. By contrast, the more advanced method is offered by the Quartz Crystal Microbalance (QCM) sensor, which uses frequency shifts to identify mass changes on its surface. The QCM sensor is suitable for in-depth laboratory analysis due to its high sensitivity and capacity to generate quantitative data, with broader dynamic range from 102 to10^7^ particles/mL. By identifying notable frequency shifts, the QCM sensor detects the viral load at lower concentrations of H5N1, performing better than the LFT but being less sensitive than the FET sensor. In addition, when compared with LFT, QCM may be less useful for on-site testing as the instrumentation is not easily portable.

Given its high sensitivity and the broadest dynamic range of all three sensors, spanning from 10 particles/mL (LOD) to 10^7^ particles/mL the FET can provide accurate readings and detect viral load at very low concentrations. The FET biosensor is perfect for confirming diagnoses in a clinical setting, even though it does require specific equipment and expertise. Its sensitivity and accuracy make it a vital tool for confirming the presence of the virus. In brief, the distinct qualities of each sensor make for a wide variety of different applications. The LFT provides rapid, on-the-spot screening yielding results quickly and clearly. For in-depth laboratory work, the QCM sensor is ideal for extensive quantitative analysis due to its high sensitivity which requires handling by a highly trained researcher due its complex hardware setting. Lastly, the extraordinary sensitivity and accurate measurement capabilities of the FET biosensor make it especially helpful in clinical settings for confirmatory testing and does not require operation by a dedicated technician. Overall, despite the speed, simplicity and affordability of LFTs, its shortcomings in terms of sensitivity and lack of quantitative output draw attention to the necessity of complementary technologies like QCM and FET sensors, which provide a more thorough assessment in both clinical and laboratory settings. Comprehensive studies of viral load are made possible by the QCM sensor's great precision in detecting mass changes, even though its portability for accurate instrumentation may be restricting. The FET biosensor's unparalleled sensitivity and quantitative precision make it an indispensable tool for confirmatory testing. In the end, this multifaceted approach can improve pandemic preparedness and control efforts by enhancing diagnostic accuracy and response times [19]. The above findings concerning the three sensors' properties are summarized in Table 2.

## Comparison of LFT for H5N1 with the LFT for SARS-CoV-2:

The SARS-CoV-2 lateral flow biosensor used for the rapid test consists of a sample pad that collects the salivary or post-nasal specimen, a conjugate pad with antibodies to gold nanoparticles targeting the SARS-CoV-2 virus, a nitrocellulose membrane with a test line featuring immobilized antibodies specific to the SARS-CoV-2 antigen with a control line of anti-species antibodies and an absorbent pad to absorb any excess. Visualization is achieved through a color change in the test and control lines. As presented in Table 3, the SARS-CoV-2 and H5N1 lateral flow biosensors jointly share high sensitivity and specificity, fast reaction times and visualization mechanisms. Despite being tailored for distinct viruses and bio-components, their shared features make them crucial instruments for promptly identifying and treating these infectious illnesses, significantly supporting public health efforts.

## Conclusions:

The present study shows that there is great potential for improving pandemic preparedness through the development and assessment of quick H5N1 virus detection techniques utilizing biosensors as preventive measures. Our research concentrates on three different biosensors, each with their own advantages and critical issues: LFT, QCM sensors and FET based electrochemical sensors. These biosensors possess distinctive features and prospective uses; their integration into public health initiatives could significantly enhance our ability to respond promptly to emerging viral hazards. The CoViD-19 pandemic has provided important insights into the need for accessibility, flexibility and scalability of diagnostic tools. By creating and testing these biosensors in time, we can enhance both early detection and prompt reactions, which can ultimately lessen the negative impact outbreaks and even prevent pandemics. The analysis we provide presents a path for the creation of cutting-edge diagnostic tools ready to uphold the protection of the world's health. A comprehensive and reliable detection system for H5N1 and other emerging infections can be built by utilizing the unique advantages that each type of biosensor has to offer. The timely need for further research and development into this field is crucial for attaining a state of preparedness and resilience against impending public health emergencies.

## Ethics approval:

Not required.

## Figures and Tables

**Table 1:** AI-based approach for each simulation and key results the measured parameters, response values, sensitivity and dynamic range of each sensor based on experimental data are focused. The dynamic range data illustrate the span between the detectable minimum and maximum concentrations, highlighting each sensor's versatility

	**LFT**	**FET**	**QCM**
Objective	To model the optical density response of LFT biosensors for detecting H5N1 virus particles in saliva.	To measure the change in current (ΔI) as an indicator of H5N1 concentration in saliva.	To quantify the relationship between virus concentration and frequency shifts for H5N1 detection in saliva.
Components and Process	The LFT uses a test strip with an H5N1-specific capture antibody-labeled conjugate zone, a test zone with anti-species antibodies and a sample pad collecting saliva on a nitrocellulose membrane. Sample migration and antibody-antigen interaction are modeled.	The FET-based sensor consists of a graphene substrate with gold electrodes. The gate is functionalized with HA antibodies specific to H5N1. Saliva samples are applied to the functionalized gate. Binding of H5N1 to the antibodies causes a change in the electrical properties, measured as a variation in the current using a potentiostat.	The QCM sensor uses a quartz crystal coated with antibodies specific to HA and NA proteins of H5N1. Saliva samples are applied to the sensor, where the binding of H5N1 causes a measurable change in the crystal's frequency. The mass change is measured as a frequency shift using a frequency counter.
Simulation Parameters	Surface tension: 0.072 N/m - Pore radius: 1x10^-6^m - Viscosity: 1.5x10^-3^ Pa.s - Association rate constant (k_a_): Specific to H5N1 antibodies - Dissociation rate constant (k_d_): Specific to H5N1 antibodies	Drain-source voltage 0.1 V - graphene channel width and length: 10^-7^ and 10^-6^ meters - graphene carrier mobility 5000cm^2^/V.s - rate constant (k_a_) 108 M^-1^s^-1^ (for viral particle binding to antibodies) - Viral particle concentrations 10^-12^M (10^4^ particles/mL) and 10^-9^ M for high concentration (10^7^ particles/mL).	Fundamental frequency (f_0_): 5 or 10 MHz - Gold electrode area: 0.2 cm^2^ - Quartz density (ρ): 2.65 g/cm^3^ - Shear modulus (µ): 2.95x10^11^ /cm.s^2^ - Low concentration: 10^4^ particles/mL - High concentration: 10^7^ particles/mL

**Table 2:** Performance summary of three sensors in detecting particle concentrations of 10^4^ and 10^7^ particles/ml

**Sensor**	**Parameter Measured**	**Response at 10^4^ particles/mL**	**Response at 10^7^ particles/mL**	**Sensitivity**	**Dynamic Range**
Lateral Flow	Optical Density (O.D.)	0.1 a.u.	0.6-0.7 a.u.	Moderate (~0.5-0.6 a.u. difference)	Effective for 10^4^ to 10^7^ particles/mL
Graphene FET	Change of Drain Current (ΔID)	24 nA	160 nA	High (136 nA difference)	Broad; effective from <10^3^ to 10^7^ particles/mL
QCM	Frequency Shift (Δf)	~0 Hz	-300 Hz	High at 10^7^ (300 Hz shift); low at 10^4^	Effective for 102 to 10^7^ particles/mL

**Table 3:** Comparison of COVID-19 and H5N1 LFT

**Feature**	**SARS-CoV-2 Lateral Flow Biosensor**	**H5N1 Lateral Flow Biosensor**
Target Virus	SARS-CoV-2	H5N1
Sample Type	Saliva/Nasal Swab	Saliva
Conjugate Pad	Antibodies conjugated to gold nanoparticles for SARS-CoV-2	Antibodies conjugated to gold nanoparticles for H5N1
Test Line	Immobilized antibodies specific to SARS-CoV-2 antigen	Immobilized antibodies specific to H5N1 antigen
Control Line	Anti-species antibodies	Anti-species antibodies
Visualization	Color change (gold nanoparticle aggregation)	Color change (gold nanoparticle aggregation)
Sensitivity	High (LOD ~ 100 particles/mL)	High (LOD ~ 100 particles/mL
Specificity	High, with cross-reactivity tests for other coronaviruses	High, with cross-reactivity tests for other influenzas
Response Time	15-30 minutes	15-30 minutes
